# The Obesity Paradox and Mortality in Older Adults: A Systematic Review

**DOI:** 10.3390/nu15071780

**Published:** 2023-04-06

**Authors:** Moustapha Dramé, Lidvine Godaert

**Affiliations:** 1 EpiCliV Research Unit, Faculty of Medicine, University of the French West Indies, 97261 Fort-de-France, France; godaert-l@ch-valenciennes.fr; 2 Department of Clinical Research and Innovation, University Hospitals of Martinique, 97261 Fort-de-France, France; 3 Department of Geriatrics, General Hospital of Valenciennes, 59300 Valenciennes, France

**Keywords:** obesity paradox, aged adults, body mass index, mortality

## Abstract

“Obesity paradox” describes the counterintuitive finding that aged overweight and obese people with a particular disease may have better outcomes than their normal weight or underweight counterparts. This systematic review was performed to summarize the publications related to the obesity paradox in older adults, to gain an in-depth understanding of this phenomenon. PubMed©, Embase©, and Scopus© were used to perform literature search for all publications up to 20 March 2022. Studies were included if they reported data from older adults on the relation between BMI and mortality. The following article types were excluded from the study: reviews, editorials, correspondence, and case reports and case series. Publication year, study setting, medical condition, study design, sample size, age, and outcome(s) were extracted. This review has been registered with PROSPERO (no. CRD42021289015). Overall, 2226 studies were identified, of which 58 were included in this systematic review. In all, 20 of the 58 studies included in this review did not find any evidence of an obesity paradox. Of these 20 studies, 16 involved patients with no specific medical condition, 1 involved patients with chronic diseases, and 2 involved patients with type 2 diabetes mellitus. Seven out of the nine studies that looked at short-term mortality found evidence of the obesity paradox. Of the 28 studies that examined longer-term mortality, 15 found evidence of the obesity paradox. In the studies that were conducted in people with a particular medical condition (*n* = 24), the obesity paradox appeared in 18 cases. Our work supports the existence of an obesity paradox, especially when comorbidities or acute medical problems are present. These findings should help guide strategies for nutritional counselling in older populations.

## 1. Introduction

Obesity, usually defined by the body mass index (BMI), is considered a public health problem, and is associated with many diseases [1,2,3]. The prevalence of obesity is high in younger adults but also in older people [4], and evidence suggests that prevalence of obesity will continue to increase [5]. The term “obesity paradox” is used to describe the counterintuitive finding that aged overweight and obese people with a particular disease may have better outcomes than their normal weight or underweight counterparts. However, there is wide heterogeneity between studies regarding the association between obesity and mortality in older adults, depending on the diseases concerned, the presence or absence of a particular disease, or the BMI level considered [6,7,8]. In aged people, body composition tends to change, and body weight tends to decrease, and some authors have suggested that fatness could be healthy [9]. Thus, it is important to confirm whether an “obesity paradox” truly exists, with a view to adapting management policies for overweight or obese old people. 

In this context, the objective of the study was to summarize the publications in the literature relating to the obesity paradox in older adults, to enhance our understanding of this phenomenon.

## 2. Methods

### 2.1. Literature Search

A preliminary check was made in PubMed©, Scopus©, Embase©, Prospero©, and the Cochrane Library© to ensure that no systematic reviews had previously been conducted on this specific topic.

A literature search was performed using PubMed©, Embase©, and Scopus© to cover all publications up to March 20, 2022. The search terms defined by the two researchers (LG, MD) included the following keywords in the title and/or the abstract: (“obesity paradox” OR “reverse epidemiology” OR “body mass index” OR BMI OR overweight OR obesity) AND (mortality OR death OR survival)). The search included studies in the French or English language and studies on human subjects, and excluded the following publication types: reviews, editorials, correspondence, and case reports and case series. A manual check was performed for potential additional studies. This systematic review was based on the Preferred Reporting Items for Systematic Reviews and Meta-Analyses (PRISMA) guidelines. This study was registered with PROSPERO (an International prospective register of systematic reviews) (number CRD42021289015), available at https://www.crd.york.ac.uk/prospero/display_record.php?ID=CRD42021289015, accessed on 20 March 2023.

### 2.2. Study Selection

Study eligibility criteria were defined a priori by the two researchers (LG, MD) within the PICOS framework. Studies were eligible if they reported data on “obesity paradox” (using body mass index as a nutritional indicator). The population was restricted to studies that included persons 65 years or older, whatever their sex, ethnicity, or living place. The intervention (exposure) was a presence of overweight or obesity as defined by the baseline BMI value. The control was those who were underweight or a normal weight. The outcome was death, whatever the timepoint. When the study was not specifically conducted in older adults, only data concerning those aged 65 years or over were taken into account (provided that the information was available). Correspondence, editorials, reviews, basic science articles, and case reports and case series were excluded.

### 2.3. Data Extraction

The Covidence systematic review software© (Veritas Health Innovation, Melbourne, Australia), available at www.covidence.org, was used to perform data analysis. After elimination of duplicates, the two researchers (LG, MD) made a blind review of titles and abstracts of all articles. When there was disagreement about whether or not to include an article, they discussed the case until consensus was reached. Overlap between studies was verified. Data extraction was realised independently by the two researchers (LG, MD), using the same extraction form. The following data were extracted: publication year, study setting, medical condition, study design, sample size, age (mean or median and their statistical dispersion parameters, when available), and outcome(s). To check whether the obesity paradox was present or not, the following information was collected: outcome(s), BMI classes, type of analysis (whether multivariable or not), statistical estimates (Hazard ratio, Odds ratio, Rate ratio, Rates) and their respective 95% confidence intervals (95% CI), and the level of significance (*p*-values).

### 2.4. Quality Assessment

The Newcastle–Ottawa Scale (NOS) [10] was used to assess the quality of included studies. This scale is composed of three quality criteria: selection (4 points), comparability (2 points), and outcome assessment (3 points). This gives a total of between 0 and 9 points. Scores of 7 or more are considered high quality studies, scores of 5–6 as moderate quality, and scores below 5 as low quality. Disagreements in scoring were resolved by a joint review of the manuscript to reach consensus.

Where possible and appropriate, some parameters were calculated from available data (e.g., mean age and/or standard deviation, rate ratio, odds ratio, etc.).

## 3. Results

**Figure 1:**
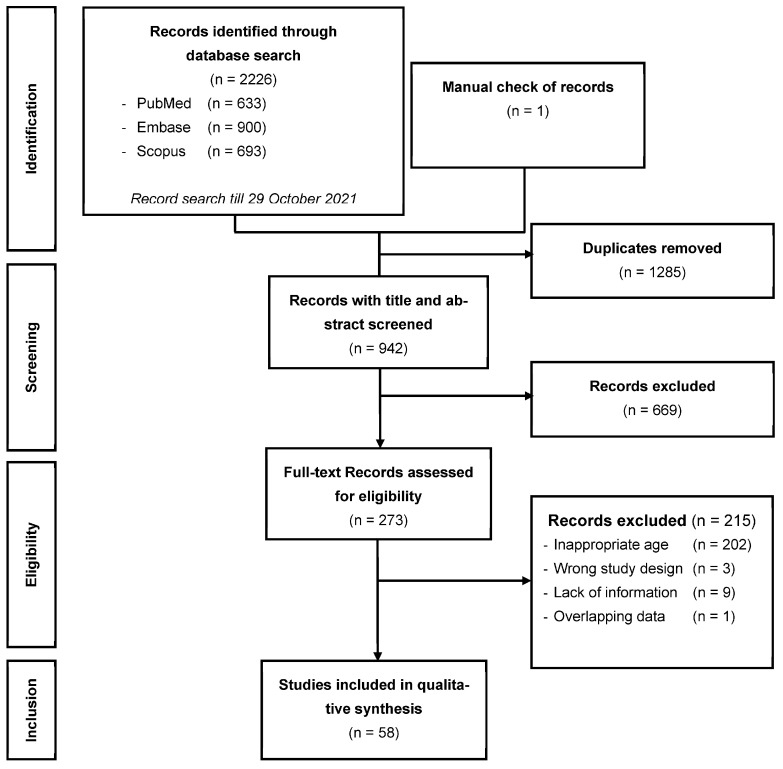
PRISMA flow diagram of the records included in the systematic review.

As shown in Figure 1, 2226 studies were identified by the literature search. Among these, 1285 duplicates were found and excluded. After checking titles and abstracts of the remaining 942 studies, 273 articles were included for full-text assessment. After full-text examination of these 273 studies, 215 were excluded for at least one of the following reasons: lack of relevant information, overlapping data, or inappropriate age of the study population. Thus, 58 studies were retained in this review.

Table 1 summarizes the characteristics of the studies included in the review. All studies were observational cohorts; 41 were prospective [11,12,13,14,15,16,17,18,19,20,21,22,23,24,25,26,27,28,29,30,31,32,33,34,35,36,37,38,39,40,41,42,43,44,45,46,47,48,49,50,51] and 17 were retrospective [52,53,54,55,56,57,58,59,60,61,62,63,64,65,66,67,68].

As shown in Table 2, 20 of the 58 studies included in this review did not find any evidence of an obesity paradox [17,27,28,29,36,39,42,43,46,47,49,50,53,56,59,62,63,65,68,69]. Of these 20 studies, 16 involved patients with no specific medical condition [17,28,29,36,39,42,43,46,47,49,50,53,56,62,65,69]. One involved patients with chronic diseases [59], and two involved patients with type 2 diabetes mellitus [27,63]. Of the 58 studies, 34 used the threshold of BMI ≥ 25.0 kg/m^2^ [11,12,14,16,19,20,21,22,24,26,30,31,32,34,38,40,41,44,45,51,52,54,55,57,58,60,66,67,68]. A further 10 studies used a threshold different from 25 kg/m^2^ and found evidence of the obesity paradox [13,18,23,25,33,35,37,48,61,64]. Regarding the time points, 9 studies looked at short-term mortality (less than 12-month mortality, ICU mortality, hospital mortality) [11,12,19,30,40,52,55,64,68]. All of these, except Yamamoto et al. [40] and Kananen et al. [68], found evidence of the obesity paradox. Of the 28 studies that examined longer-term mortality (time point ≥ 5 years) [13,14,15,20,22,27,28,32,34,36,37,38,39,42,44,45,46,49,53,56,57,58,59,60,61,62,63,66,67], 15 (54%) found evidence of the obesity paradox [13,14,20,22,32,34,37,38,44,45,57,58,60,61,66,67]. In the studies that were conducted in people with a particular medical condition (*n* = 24) [11,12,14,16,18,19,21,24,25,26,27,37,40,44,52,54,55,58,59,60,63,64,66,68], the obesity paradox appeared in 18 (75%) cases [11,12,14,16,18,19,21,24,25,26,37,40,44,52,54,55,58,60,64,66]. In the studies that were carried out among people with no specific medical condition (*n* = 34) [13,15,17,20,22,23,28,29,30,31,32,33,34,35,36,38,39,41,42,43,45,46,47,48,49,50,51,53,56,57,61,62,65,67], the obesity paradox appeared in 17 (50%) cases [22,23,30,31,32,33,34,35,38,41,45,48,51,57,61,67].

An appendix provides detailed information of the analyses and results of the relationship between BMI and mortality in aged adults. Of the analyses tested for the existence of an obesity paradox, 48 were adjusted for confounders, and 10 were unadjusted analyses (see Supplementary Materials).

The quality of the included studies, as assessed using the NOS, was considered high for all 58 studies (Table 3).

## 4. Discussion

In this systematic review of studies exploring the relationship between BMI and mortality in patients aged 65 years or older, 28 out of the 58 studies included observed longer survival in patients with a BMI ≥ 25 kg/m^2^ (the so-called obesity paradox) [11,12,14,16,19,20,21,22,24,26,30,31,32,34,38,40,41,44,45,51,52,54,55,57,58,60,66,67]. Among these 28 studies, 16 involved patients with a specific or acute medical condition [11,12,14,16,19,21,24,26,40,44,52,54,55,58,60,66]. Seven studies found improved survival in overweight and obese older people when focussing on short-term mortality [11,12,19,30,52,55,64,70]. One showed increased survival only in the oldest patients [25]. Two showed increased survival only in men [14,44]. Of the 23 studies that did not observe an obesity paradox [14,15,17,25,27,28,29,36,39,40,42,43,46,47,49,50,53,56,59,62,63,65,68], 7 involved populations selected according to the presence of a particular medical condition [14,25,27,40,59,63,68].

Nearly two-thirds of the studies included in this work report better survival in overweight or obese older people. Several factors may influence the relationship between obesity and survival in the older population, including age, degree of obesity, presence or absence of comorbidities, and occurrence of an acute event.

Regarding age, the studies in this review that failed to show better survival in overweight or obese individuals included populations that were, on average, younger than those demonstrating an obesity paradox. Wu et al. [25], in their study of the impact of age on the association between BMI and all-cause mortality in patients with atrial fibrillation, found better survival in overweight or obese patients aged 75 years or older but not in patients aged between 65 and 74 years. Observations made in older populations must therefore take into account the intrinsic characteristics of the survivors. For the same BMI, patient profiles can be different, and this profile can influence survival. For instance, body composition may differ due to ethnicity, sex, or advancing age [71,72]. BMI does not provide information on body composition, and is less correlated with percentage of body mass or fat mass index, especially in younger people [72]. Abdominal obesity has direct metabolic consequences (adipose tissue inflammation, dysglycaemia, alteration of blood pressure regulation, etc.). Conversely, subcutaneous fat accumulation in the hips, for example, appears to have benign effects on cardiovascular risk. Other indicators, such as waist circumference or waist-to-hip ratio, are strongly associated with higher mortality risk [73,74]. Taking only BMI into account does not make it possible to differentiate between these situations [9]. In all studies included in this work, BMI was defined as an obesity index. If obesity is defined by “body adiposity”, BMI level is probably not the best criterion [75]. The term “BMI paradox” may be more appropriate than “obesity paradox”, as suggested by Antonopoulos et al. [9].

Obesity is a factor associated with higher mortality in younger populations [76,77,78], but it is also associated with an increased risk of developing and dying from a number of diseases [3], such as cancer [79,80], Some authors point to the obesity-related cellular and immune changes that make obese people more vulnerable, including an increased risk of infections [1]. Older obese people could be considered constitutionally more robust as they have survived the risk factor of obesity into adulthood. The degree of obesity could also be a factor. In this review, not all authors differentiated between different classes of obesity. However, the positive effect on survival in cases of overweight and obesity was not found for morbid obesity (BMI ≥ 35.0 kg/m²) in 5 studies [11,32,57,58,66]. Furthermore, weight is not a reflection of body composition, in particular the muscle mass/fat mass ratio. Loss of muscle mass and strength (sarcopenia) is a factor associated with an increased risk of death. Tian et al. reported that obese people with sarcopenia have a higher risk of death than obese people without sarcopenia [81]. Obese people may be less frequently sarcopenic than non-obese people. In 1493 subjects aged 65 years or more (median age 74 ± 11 years), Sousa-Santos et al. [82] found a prevalence of 0.8% of obese sarcopenic individuals versus 11.6% of sarcopenic individuals of all BMI status.

The presence of a chronic pathology or an acute event may also influence survival. In this review, 20 studies [11,12,14,16,18,19,21,24,25,26,37,40,44,52,54,55,58,60,64,66] of the 38 which found a favourable effect of overweight or obesity on survival involved patients with a particular chronic condition or facing a specific medical event. This finding suggests that even moderately overweight older individuals with chronic disease or acute medical events have better survival. Obesity in older people with a chronic disease could be a sign of greater robustness or higher reserves (better appetite, less risk of undernutrition). Overweight or obese older subjects would be less undernourished than the general older population. Cereda et al. [83], in their meta-analysis of the prevalence of undernutrition in an older population, found a prevalence of undernutrition ranging from 3.1 to 29.4%, depending on the setting. Sousa-Santos et al. [84] showed that 6% of obese elderly subjects (BMI ≥ 30 kg/m^2^) were also undernourished or at risk of undernutrition. In the event of an acute event, obese elderly people may have a better chance of survival, particularly because of their greater functional reserves. This observation is also made in younger obese or overweight subjects. Akinnusi et al. [85] show in their meta-analysis of patients admitted to intensive care that obese subjects have a similar mortality to non-obese subjects. In 2013, the meta-analysis by Flegal et al. [76] confirmed in a population without any particular pathology that overweight people (BMI > 25 kg/m²) (all types of obesity and all ages) had a higher overall mortality rate, whatever the cause. However, mildly overweight people (BMI ≥ 25 and <30 kg/m²) had lower all-cause mortality than normal weight people (BMI < 25 kg/m²). Thus, this advantage was found regardless of age.

Several mechanisms could explain “obesity paradox”. Probably, there are “good adipose tissues” in elderly subjects. In the literature, overweight or obesity, defined by high level of BMI, is shown to have positive influence on prothrombotic factors, production of certain cytokines, or NT-proBNP levels. Adipokine produced by adipose tissue seems to be cardioprotective [86]. Obesity could have a protective effect against progression or consequences of some chronic diseases. High BMI could also reflect better nutritional status and adequate muscle reserves. Casas-Vara et al. [87] showed better nutritional status in overweight or obese elderly people with heart failure.

Our systematic review has limitations. Although the WHO has proposed thresholds for BMI, the authors used different thresholds in their respective studies. In addition, the outcomes were also different between the studies. This made it difficult to compare the studies, and precluded meta-analysis. The age variable was missing in 14.0% of cases (8/57).

However, this work covers a large number of studies, totalling more than 1,120,000 people aged 65 years or over, with varying medical conditions and in different settings. The follow-up time of the studies ranged from 30 days to 156 months (even though the majority of studies have a long-term follow-up). These differences in follow-up time may make comparison difficult. In addition, there is no information on BMI variation over time, especially for studies with long-term follow-up. Weight loss or gain between baseline measurement and death could have a significant impact. The fact that only studies conducted in subjects aged 65 years or older were selected gives a certain homogeneity to this systematic review in terms of population. Finally, all studies were evaluated for methodological quality using the NOS, and were found to be of high quality.

## 5. Conclusions

The findings of this systematic review are in favour of the existence of an obesity paradox, which could more specifically concern older subjects with a comorbidity and/or experiencing an acute event. Nevertheless, because BMI does not reflect body composition, the term “BMI paradox” would be more appropriate. The influence of the level of BMI remains unclear. These findings should help guide strategies for nutritional counselling in the older population.

## Figures and Tables

**Table 1:** Description of the studies included in the present systematic review.

Author, Year	Country	Study Design	Study Setting	Medical Condition	Sample Size	Age (Years) Mean ± SD
Kananen, 2022 [68]	Sweden	Retrospective cohort	Hospital, Geriatrics	COVID-19	1409	77 [65–104] ^♣^
Amin, 2021 [11]	USA	Prospective cohort	Hospital, Surgery	Hip fracture	52,729	x ± x
Danninger, 2021 [52]	USA	Retrospective cohort	Hospital, ICU	Sepsis	8707	x ± x
El Moheb, 2021 [12]	USA	Prospective cohort	Hospital, Surgery	Emergent surgery	78,704	75 ± x
Lin, 2021 [13]	Taiwan	Prospective cohort	Community	None specific	81,221	74 ± 6
Martinez-Tapia, 2021 [14]	France	Prospective cohort	Hospital, Geriatrics	Cancer	2071	81 ± 6
Lai, 2020 [15]	Taiwan	Prospective cohort	LTCF	None specific	182	79 ± 8
Schneider, 2020 [16]	Germany	Prospective cohort	Hospital, Neurosurgery	Glioblastoma	110	72 [65–86] ^♣^
Seino, 2020 [53]	Japan	Retrospective cohort	Community	None specific	1977	72 ± 6 *
Nishida, 2019 [17]	Japan	Prospective cohort	Community	None specific	1229	74 ± 5
Om, 2019 [18]	Korea	Prospective cohort	Hospital, Cardiology	Aortic stenosis	379	79 ± x *
Tokarek, 2019 [54]	Poland	Retrospective cohort	Hospital, Cardiology	TAVI patients	147	82 [x–x] ^♣^
Yoshihisa, 2019 [19]	Japan	Prospective cohort	Hospital, Cardiology	Acute heart failure	2410	x ± x
Crotti, 2018 [20]	Italy	Prospective cohort	Community	None specific	4970	72 ± 5
De Palma, 2018 [21]	Sweden	Prospective cohort	Hospital, Cardiology	TAVI patients	492	83 ± 6
Keller, 2018 [55]	Germany	Retrospective cohort	Hospital, Cardiology	AMI	122,607	80 ± x
Kim, 2018 [22]	Korea	Prospective cohort	Community	None specific	170,639	72 ± 5
Lee, 2018 [56]	Korea	Retrospective cohort	Community	None specific	11,844	72 ± 5
Lv, 2018 [23]	China	Prospective cohort	Community	None specific	4361	92 ± 8
de Souto Barreto, 2017 [24]	France	Prospective cohort	Nursing home	Dementia	3741	86 ± 8
Wu, 2017 [25]	China	Prospective cohort	Hospital, ED	Atrial fibrillation	1321	x ± x
Cheng, 2016 [57]	USA	Retrospective cohort	Community	None specific	4565	74 ± 5
Flodin, 2016 [26]	Sweden	Prospective cohort	Hospital	Hip fracture	843	82 ± 7
Calabia, 2015 [58]	Spain	Retrospective cohort	Hospital, Nephrology	Haemodialysis	3978	75 ± 6
Kim, 2015 [59]	Korea	Retrospective cohort	Community	Chronic diseases	x	x ± x
Kubota, 2015 [60]	Japan	Retrospective cohort	Community	T2DM	16,304 ^#^	x ± x
Kuo, 2015 [27]	Taiwan	Prospective cohort	Outpatients	T2DM	x	x ± x
Shil Hong, 2015 [61]	Korea	Retrospective cohort	Community	None specific	1000	76 ± 9
Buys, 2014 [28]	USA	Prospective cohort	Community	None specific	1257	75 ± 7
Clark, 2014 [62]	USA/Nigeria	Retrospective cohort	Community	None specific	2466	77 ± 5 *
Ford, 2014 [29]	USA	Prospective Cohort	Community	None specific	2995	81 ± 4
Lang, 2014 [30]	France	Prospective cohort	Hospital, ED	None specific	1306	85 ± 6
Lee, 2014 [31]	Korea	Prospective cohort	Community	None specific	11,844	73 ± 7
Murphy, 2014 [63]	Iceland	Retrospective cohort	Community	T2DM	637	77 [66–96] ^♠^
Wu, 2014 [32]	Taiwan	Prospective cohort	Community	None specific	77,541	73 ± 7
Yamauchi, 2014 [64]	Japan	Retrospective cohort	Hospital, Pulmonology	COPD	263,940	78 ± 7
Chen, 2013 [33]	Taiwan	Prospective cohort	Veterans	None specific	1257	83 ± 5
Dahl, 2013 [34]	Sweden	Prospective cohort	Community	None specific	882	80 ± 6
Nakazawa, 2013 [35]	Japan	Prospective cohort	Nursing home	None specific	8510	84 ± 8
Takata, 2013 [36]	Japan	Prospective cohort	Community	None specific	675	80 ± 0
Tseng, 2013 [37]	Taiwan	Prospective cohort	Community	T2DM	34,825	x ± x
Veronese, 2013 [38]	Italy	Prospective cohort	Nursing home	None specific	181	81 ± 8
Woo, 2013 [39]	China	Prospective cohort	Community	None specific	4000	73 ± 5
Yamamoto, 2013 [40]	France	Prospective cohort	Hospital, Cardiology	TAVI patients	3072	83 ± 7
Zekry, 2013 [41]	Switzerland	Prospective cohort	Hospital, Geriatric	None specific	444	85 ± 7
de Hollander, 2012 [42]	Netherlands	Prospective cohort	Community	None specific	1980	73 ± 2
Kvamme, 2012 [43]	Norway	Prospective cohort	Community	None specific	16,711	73 ± 5
Mihel, 2012 [44]	Croatia	Prospective cohort	Community	Hypertension	2507	x ± x
Tsai, 2012 [65]	Taiwan	Retrospective cohort	Community	None specific	2892	x ± x
Cereda, 2011 [45]	Italy	Prospective cohort	LTCF	None specific	533	84 ± 8
Berraho, 2010 [46]	France	Prospective cohort	Community	None specific	3646	75 ± 7
Han, 2010 [47]	Korea	Prospective cohort	Community	None specific	877	75 ± 8
Kitamura, 2010 [48]	Japan	Prospective cohort	Home care	None specific	205	84 ± 8
Lea, 2009 [66]	USA	Retrospective cohort	Hospital, Cardiology	AMI	74,167	77 ± x *
Luchsinger, 2008 [49]	USA	Prospective cohort	Community	None specific	1372	78 ± 6
Locher, 2007 [50]	USA	Prospective cohort	Community	None specific	983	75 ± 7
Takata, 2007 [51]	Japan	Prospective cohort	Community	None specific	697	80 ± 0
Grabowski, 2001 [67]	USA	Retrospective cohort	Community	None specific	7527	77 ± 6

SD: Standard deviation; ICU: Intensive care unit; ED: Emergency department; TAVI: Transcatheter Aortic Valve Implementation; COPD: Chronic Obstructive Pulmonary Disease; AMI: Acute Myocardial Infarction; T2DM: Type 2 Diabetes Mellitus; LTCF: Long-term care facility. x: Missing information; ^#^: Person-years; *: Pooled mean and/or standard deviation have been calculated with the information available in these articles; ♣: Median [range]; ♠: Mean [range].

**Table 2:** Outcomes and association between body mass index group and mortality in aged adults.

Author(s), Year	Age (Mean ± SD)	Medical Condition	Outcome	Obesity Paradox	BMI Thresholds ^#^ (kg/m^2^)
Kananen, 2022 [68]	x ± x	COVID-19	In-hospital mortality	No	18.5 < BMI < 25.0
Amin, 2021 [11]	x ± x	Hip fracture	30-day mortality	Yes	BMI ≥ 25.0 (No, if BMI > 40.0)
Danninger, 2021 [52]	x ± x	Sepsis	ICU mortality	Yes	BMI ≥ 30.0
El Moheb, 2021 [12]	75 ± x	Emergent Surgery	30-day mortality	Yes	BMI ≥ 25.0
Lin, 2021 [13]	74 ± 6	None specific	84-month mortality	Yes	BMI ≥ 24.0
Martinez-Tapia, 2021 [14]	81 ± 6	Cancer	12-month mortality (men)	Yes	BMI ≥ 30.0
			12-month mortality (women)	No	
			60-month mortality (men)	Yes	BMI ≥ 30.0
			60-month mortality (women)	Yes	BMI ≥ 30.0
Lai, 2020 [15]	79 ± 8	None specific	72-month mortality	No	
Schneider, 2020 [16]	72 ± x	Glioblastoma	12-month mortality	Yes	BMI ≥ 30.0
Seino, 2020 [53]	72 ± 6	None specific	All-cause mortality (men)	No	
			All-cause mortality (women)	No	
Nishida, 2019 [17]	74 ± 5	None specific	36-month mortality	No	
Om, 2019 [18]	79 ± x	Aortic stenosis	12-month mortality	Yes	BMI ≥ 24.9
Tokarek, 2019 [54]	82 ± x	TAVI patients	12-month survival	Yes	BMI ≥ 30.0
Yoshihisa, 2019 [19]	x ± x	AHF	In-hospital mortality	Yes	BMI ≥ 25.0
Crotti, 2018 [20]	72 ± 5	None specific	68-month mortality	Yes	BMI ≥ 25.0 (No, if BMI > 30.0)
			68-month CVD mortality	No	
			68-month cancer mortality	No	
De Palma, 2018 [21]	83 ± 6	TAVI patients	12-month mortality	Yes	BMI ≥ 25.0
			50-month mortality	Yes	BMI ≥ 25.0
Keller, 2018 [55]	80 ± x	AMI	In-hospital mortality	Yes	BMI ≥ 30.0
Kim, 2018 [22]	72 ± 5	None specific	60-month mortality	Yes	BMI ≥ 25.0 (No, if BMI > 27.5)
Lee, 2018 [56]	72 ± 5	None specific	60-month mortality	No	
Lv, 2018 [23]	92 ± 8	None specific	36-month mortality	Yes	BMI ≥ 18.5
De Souto Barreto, 2017 [24]	86 ± 8	Dementia	18-month mortality (dementia)	Yes	BMI ≥ 25.0
			18-month mortality (without dementia)	Yes	BMI ≥ 25.0
Wu, 2017 [25]	x ± x	Atrial fibrillation	12-month mortality (65–74 years)	No	
			12-month mortality (≥75 years)	Yes	BMI ≥ 24.0
Cheng, 2016 [57]	74 ± 5	None specific	132-month mortality	Yes	BMI ≥ 25.0 (No, if BMI ≥ 35.0)
		Diabetes		Yes	BMI ≥ 25.0 (No, if BMI ≥ 35.0)
		Hypertension		Yes	BMI ≥ 25.0 (No, if BMI ≥ 35.0)
		Dyslipidaemia		Yes	BMI ≥ 25.0 (No, if BMI ≥ 35.0)
Flodin, 2016 [26]	82 ± 7	Hip fracture	12-month survival	Yes	BMI > 26.0
Calabia, 2015 [58]	75 ± 6	Haemodialysis	120-month mortality	Yes	BMI = 30.0–34.9 (No, if BMI = 27.5–29.9 or BMI ≥ 35.0)
Kim, 2015 [59]	x ± x	Chronic diseases	108-month mortality	No	
Kubota, 2015 [60]	x ± x	T2DM	132-month ID mortality	Yes	BMI ≥ 25.0
Kuo, 2015 [27]	x ± x	T2DM	66-month mortality	No	
Shil hong, 2015 [61]	76 ± 9	None specific	72-month mortality	Yes	BMI ≥ 23.8
Buys, 2014 [28]	75 ± 7	None specific	102-month mortality	No	
Clark, 2014 [62]	77 ± 5	None specific	120-month mortality (Africans)	No	
			120-month mortality (African Americans)	No	
Ford, 2014 [29]	81 ± 4	None specific	40-month mortality	No	
Lang, 2014 [30]	85 ± 6	None specific	6-week mortality	Yes	BMI ≥ 30.0
			12-month mortality	Yes	BMI ≥ 25.0
			24-month mortality	Yes	BMI ≥ 25.0
Lee, 2014 [31]	73 ± 7	None specific	36-month mortality	Yes	BMI ≥ 25.0 (No, if BMI ≥ 30.0)
Murphy, 2014 [63]	77 ± x	T2DM	84-month mortality	No	
Wu, 2014 [32]	73 ± 7	None specific	60-month mortality	Yes	BMI ≥ 25.0 (No, if BMI ≥ 35.0)
			60-month CVD mortality		BMI ≥ 25.0 (No, if BMI ≥ 30.0)
Yamauchi, 2014 [64]	78 ± 7	COPD	In-hospital mortality	Yes	BMI ≥ 23.0
Chen, 2013 [33]	83 ± 5	None specific	18-month mortality	Yes	BMI ≥ 23.0
Dahl, 2013 [34]	80 ± 6	None specific	216-month mortality	Yes	BMI ≥ 25.0 (No, if BMI ≥ 30.0)
Nakazawa, 2013 [35]	84 ± 8	None specific	12-month mortality	Yes	BMI ≥ 23.6
Takata, 2013 [36]	80 ± 0	None specific	144-month mortality	No	
			144-month CVD mortality	No	
			144-month cancer mortality	No	
Tseng, 2013 [37]	x ± x	T2DM	144-month mortality	Yes	BMI ≥ 23.0
Veronese, 2013 [38]	81 ± 8	None specific	60-month	Yes	BMI ≥ 30.0
Woo, 2013 [39]	73 ± 5	None specific	84-month mortality	No	
Yamamoto, 2013 [40]	83 ± 7	TAVI patients	30-day mortality	No	
			12-month mortality	Yes	BMI ≥ 25.0
Zekry, 2013 [41]	85 ± 7	None specific	48-month mortality	Yes	BMI ≥ 30.0
de Hollander, 2012 [42]	73 ± 2	None specific	120-month mortality	No	
Kvamme, 2012 [43]	73 ± 5	None specific	12-month mortality (men)	No	
			12-month mortality (women)	No	
		Respiratory diseases	12-month mortality (men)	No	
			12-month mortality (women)	No	
		CVD	12-month mortality (men)	No	
			12-month mortality (women)	No	
		Cancer	12-month mortality (men)	No	
			12-month mortality (women)	No	
Mihel, 2012 [44]	x ± x	Hypertension	60-month mortality (men)	Yes	BMI ≥ 30.0
			60-month mortality (women)	No	
Tsai, 2012 [65]	x ± x	None specific	48-month mortality (65–74 y; men)	No	
			48-month mortality (≥75 y; men)	No	
			48-month mortality (65–74 y; women)	No	
			48-month mortality (≥75 y; women)	No	
Cereda, 2011 [45]	84 ± 8	None specific	72-month mortality	Yes	BMI ≥ 25.0
Berraho, 2010 [46]	75 ± 7	None specific	156-month mortality	No	
Han, 2010 [47]	75 ± 8	None specific	42-month mortality	No	
Kitamura, 2010 [48]	84 ± 8	None specific	24-month mortality	Yes	BMI ≥ 17.1
Lea, 2009 [66]	77 ± x	AMI	125-month mortality	Yes	BMI ≥ 25.0 (No, if BMI > 40.0)
Luchsinger, 2008 [49]	78 ± 6	None specific	144-month mortality	No	
Locher, 2007 [50]	75 ± 7	None specific	36-month mortality	No	
Takata, 2007 [51]	80 ± 0	None specific	48-month mortality	Yes	BMI ≥ 25.0
			48-month CVD mortality	No	
			48-month cancer mortality	No	
Grabowski, 2001 [67]	77 ± 6	None specific	96-month mortality	Yes	BMI ≥ 28.5

^#^ BMI thresholds at which an obesity paradox was demonstrated. SD: Standard deviation; ICU: Intensive Care Unit; TAVI: Transcatheter Aortic Valve Implementation; COPD: Chronic Obstructive Pulmonary Disease; AHF: Acute Heart Failure; AMI: Acute Myocardial Infarction; T2DM: Type 2 Diabetes Mellitus; CVD: Cardiovascular disease; y, years. x: Missing information.

**Table 3:** Quality assessment of the different studies included in this systematic review, using the Newcastle–Ottawa scale (NOS).

Author, Year	Study Design	Selection	Comparability	Outcome	Total Score	Quality Rating
Kananen, 2022 [68]	Retrospective cohort	****	**	***	9	High
Amin, 2021 [11]	Prospective cohort	****	**	***	9	High
Danninger, 2021 [52]	Retrospective cohort	****	**	***	9	High
El Moheb, 2021 [12]	Prospective cohort	****	**	***	9	High
Lin, 2021 [13]	Prospective cohort	***	**	***	8	High
Martinez-Tapia, 2021 [14]	Prospective cohort	****	**	***	9	High
Lai, 2020 [15]	Prospective cohort	****	**	***	9	High
Schneider, 2020 [16]	Prospective cohort	****	**	***	9	High
Seino, 2020 [53]	Retrospective cohort	****	**	***	9	High
Nishida, 2019 [17]	Prospective cohort	****	**	***	9	High
Om, 2019 [18]	Prospective cohort	****	*	***	8	High
Tokarek, 2019 [54]	Retrospective cohort	****	*	***	8	High
Yoshihisa, 2019 [19]	Prospective cohort	****	*	***	8	High
Crotti, 2018 [20]	Prospective cohort	****	**	***	9	High
De Palma, 2018 [21]	Prospective cohort	****	*	***	8	High
Keller, 2018 [55]	Retrospective cohort	****	*	***	8	High
Kim, 2018 [22]	Prospective cohort	****	**	***	9	High
Lee, 2018 [56]	Retrospective cohort	****	**	***	9	High
Lv, 2018 [23]	Prospective cohort	****	**	***	9	High
de Souto Barreto, 2017 [24]	Prospective cohort	****	**	***	9	High
Wu, 2017 [25]	Prospective cohort	****	**	***	9	High
Cheng, 2016 [57]	Retrospective cohort	****	**	***	9	High
Flodin, 2016 [26]	Prospective cohort	****	**	***	9	High
Calabia, 2015 [58]	Retrospective cohort	****	**	***	9	High
Kim, 2015 [59]	Retrospective cohort	****	**	***	9	High
Kubota, 2015 [60]	Retrospective study	****	**	***	9	High
Kuo, 2015 [27]	Prospective cohort	****	*	***	8	High
Shil Hong, 2015 [61]	Retrospective cohort	****	**	***	9	High
Buys, 2014 [28]	Prospective cohort	***	**	***	8	High
Clark, 2014 [62]	Retrospective cohort	****	**	***	9	High
Ford, 2014 [29]	Prospective cohort	***	**	***	8	High
Lang, 2014 [30]	Prospective cohort	****	**	***	9	High
Lee, 2014 [31]	Prospective cohort	****	**	***	9	High
Murphy, 2014 [63]	Retrospective cohort	****	**	***	9	High
Wu, 2014 [32]	Prospective cohort	****	**	***	9	High
Yamauchi, 2014 [64]	Retrospective cohort	****	**	***	9	High
Chen, 2013 [33]	Prospective cohort	***	**	***	8	High
Dahl, 2013 [34]	Prospective cohort	***	**	***	8	High
Nakazawa, 2013 [35]	Prospective cohort	****	**	***	9	High
Takata, 2013 [36]	Prospective cohort	***	**	***	8	High
Tseng, 2013 [37]	Prospective cohort	****	**	***	9	High
Veronese, 2013 [38]	Prospective cohort	***	**	***	8	High
Woo, 2013 [39]	Prospective cohort	****	**	***	9	High
Yamamoto, 2013 [40]	Prospective cohort	****	**	***	9	High
Zekry, 2013 [41]	Prospective cohort	****	**	***	9	High
de Hollander, 2012 [42]	Prospective cohort	***	**	***	8	High
Kvamme, 2012 [43]	Prospective cohort	****	**	***	9	High
Mihel, 2012 [44]	Prospective cohort	***	*	***	7	High
Tsai, 2012 [65]	Retrospective cohort	****	**	***	9	High
Cereda, 2011 [45]	Prospective cohort	***	**	***	8	High
Berraho, 2010 [46]	Prospective cohort	****	**	***	9	High
Han, 2010 [47]	Prospective cohort	****	**	***	9	High
Kitamura, 2010 [48]	Prospective cohort	****	**	***	9	High
Lea, 2009 [66]	Retrospective cohort	****	**	***	9	High
Luchsinger, 2008 [49]	Prospective cohort	****	**	***	9	High
Locher, 2007 [50]	Prospective cohort	****	**	***	9	High
Takata, 2007 [51]	Prospective cohort	****	**	***	9	High
Grabowski, 2001 [67]	Retrospective cohort	****	**	***	9	High

Each star is equal to one point. The sum of the stars gives the total score of the NOS. NOS score of ≥7 were considered as high quality studies, NOS score of 5–6 as moderate quality, and NOS Scores less than 5 as low quality.

## Data Availability

Data could be made available on reasonable request at moustapha.drame@chu-martinique.fr.

## Supplementary Materials

The following supporting information can be downloaded at: https://www.mdpi.com/article/10.3390/nu15071780/s1, Table S1: Outcome and results of association between body mass index groups and mortality in aged adults (detailed information).
