# Supplementary material for: The Obesity Paradox and Mortality in Older Adults: A Systematic Review
Source: Nutrients. 2023 Apr 6;15(7):1780. doi: 10.3390/nu15071780 (PMC10096985; doi:10.3390/nu15071780)
Supplement: Supplementary file 1 [file nutrients-15-01780-s001.zip › nutrients-2308954-supplementary.pdf]

Table S1: Outcome and results of association between body mass index groups and mortality in aged adults (detailed information)

Author, year	Outcome	Medical condition	Analysis	Results: BMI Classes (kg/m²)	Results: Estimates (95% CI)	Results: p	Obesity paradox
Kananen, 2022 [68]	In-hospital mortality	Covid-19	Adjusted OR	<18.5	2.30 (1.17–4.31)	.01	No
				18.5–24.9	Reference		
				25.0–29.9	1.05 (0.63–1.70)	.86	
				≥ 30.0	1.23 (0.57–2.45)	.58	
Amin, 2021 [11]	30-day mortality	Hip fracture	Adjusted HR	<18.5	1.54 (1.06–2.24)	<.0001	Yes
				18.5–24.9	Reference		
				25.0–29.9	0.69 (0.62–0.77)	<.0001	
				30.0–34.9	0.62 (0.51–0.74)	<.0001	
				35.0–39.9	0.69 (0.50–0.95)	.02	
				40.0–49.9	0.65 (0.38–1.13)	.13	
				≥ 50.0	1.15 (0.51–2.60)	.73	
Danninger, 2021 [52]	ICU mortality	Sepsis	Adjusted OR	<18.5	1.57 (1.16–2.12)	.003	Yes
				18.5–24.9	Reference		
				25.0–29.9	0.88 (0.73–1.06)	.18	
				≥ 30.0	0.82 (0.68–0.98)	.03	
El Moheb, 2021 [12]	30-day mortality	Emergent surgery	Adjusted HR	18.5–24.9	Reference		Yes
				25.0–29.9	0.80 (0.75–0.84)	<.05	
				30.0–34.9	0.80 (0.74–0.85)	<.05	

				35.0–39.9	0.70 (0.63–0.77)	<.05	
				≥ 40.0	0.82 (0.74–0.91)	<.05	
Lin, 2021 [13]	84-month mortality	None specific	Adjusted HR	<18.5	1.95 (1.79–2.12)	<.01	Yes
				18.5–23.9	Ref.		
				24.0–26.9	0.75 (0.71–0.80)	<.01	
				≥ 27.0	0.74 (0.69–0.79)	<.01	
Martinez-Tapia,2021 [14]	12-month mortality	Cancer	Unadjusted HR (Men)	<22.5	1.64 (1.31-2.07)	<.0001	Yes
				22.5–24.9	Reference		
				25.0–29.9	0.88 (0.69-1.11)		
				≥ 30.0	0.70 (0.50-0.98)		
			Unadjusted HR (Women)	<22.5	1.65 (1.25-2.18)		Yes
				22.5–24.9	Reference		
				25.0–29.9	1.03 (0.76-1.40)		
				≥ 30.0	0.72 (0.49-1.06)		
	60-month mortality		Unadjusted HR (Men)	<22.5	1.51 (1.25-1.81)		Yes
				22.5–24.9	Reference		
				25.0–29.9	0.84 (0.70-1.01)		
				≥ 30.0	0.74 (0.58-0.95)		
			Unadjusted HR (Women)	<22.5	1.31 (0.07-1.61)	<.0001	Yes
				22.5–24.9	Reference		
				25.0–29.9	0.95 (0.77-1.18)		
				≥ 30.0	0.60 (0.46-0.79)		
Lai, 2020 [15]	72-month	None specific	Adjusted HR	<18.5	1.80 (1.11-2.94)	.02	No

	mortality			18.5–23.9	Reference		
				24.0–26.9	1.23 (0.68-2.21)	.50	
				≥ 27.0	0.39 (0.14-1.07)	.07	
Schneider, 2020 [16]	12-month mortality	Glioblastoma	Adjusted OR	<30.0	3.10 (1.10–9.20)	.04	Yes
				≥ 30.0	Reference		
Seino, 2020 [53]	All-cause mortality	None specific	Adjusted HR (Men)	10th percentile (19.8)	1.30 (0.92–1.83)	NS	No
				50th percentile (23.3)	Reference		
				90th percentile (27.2)	0.65 (0.41–1.03)	NS	
			Adjusted HR (Women)	10th percentile (18.9)	1.87 (1.18–2.95)	S	No
				50th percentile (22.8)	Reference		
				90th percentile (27.3)	0.88 (0.54–1.42)	NS	
Nishida, 2019 [17]	36-month mortality	None specific	Adjusted HR	<18.5	1.55 (0.41–5.85)	.52	No
				18.5–23.0	Reference		
				23.0–27.5	1.00 (0.42–2.39)	1.00	
				≥ 27.5	0.96 (0.12–7.67)	.97	
Om, 2019 [18]	12-month mortality	Aortic stenosis	Unadjusted rates	≤22.3	19%	.006	Yes
				22.4–24.8	10%		

				≥ 24.9	6%		
Tokarek, 2019 [54]	12-month survival	TAVI patients	Unadjusted HR	18.5–24.9	Reference		Yes
				25.0–29.9	2.26 (0.91–5.60)	.008	
				≥ 30.0	3.86 (1.43–10.46)		
Yoshihisa, 2019 [19]	In-hospital mortality	AHF	Unadjusted OR	≥ 25.0	Reference		Yes
				< 25.0	3.49 (2.05–5.97)	<.001	
Crotti, 2018 [20]	68-month mortality	None specific	Adjusted HR	18.5–24.9	Reference		Yes
				25.0–29.9	0.76 (0.63–0.92)	<.05	
				30.0–39.9	0.85 (0.69–1.04)	NS	
				≥ 40.0	1.53 (0.92–2.52)	NS	
	68-month CVD mortality			18.5–24.9	Reference		No
				25.0–29.9	0.81 (0.61–1.08)	NS	
				30.0–39.9	0.92 (0.67–1.26)	NS	
				≥ 40.0	1.14 (0.45–2.88)	NS	
	68-month cancer mortality			18.5–24.9	Reference		No
				25.0–29.9	0.99 (0.70–1.39)	NS	
				30.0–39.9	1.07 (0.74–1.54)	NS	
				≥ 40.0	2.91 (1.43–5.94)	<.05	
De Palma, 2018 [21]	12-month mortality	TAVI patients	Unadjusted HR	≥ 25.0	0.63 (0.45–0.86)	.005	Yes
				< 25.0	Reference		
	50-month mortality		Unadjusted HR	≥ 25.0	0.68 (0.50–0.93)	.02	Yes
				< 25.0	Reference		
Keller, 2018 [55]		AMI	Unadjusted	<30	Reference		Yes

	In-hospital mortality		OR#	30–34.9	0.41 (0.36–0.48)	<.0001	
				35–39.9	0.59 (0.47–0.74)	<.0001	
				≥ 40	0.73 (0.54–0.98)	.04	
Kim, 2018 [22]	60-month mortality	None specific	Adjusted HR (men)	16.0–17.4	2.87 (2.60-3.18)	<.0001	Yes
				17.5–19.9	1.84 (1.74-1.95)	<.0001	
				20.0–22.4	1.25 (1.119-1.31)	<.0001	
				22.5–24.9	Reference		
				25.0–27.4	0.86 (0.81-0.91)	<.0001	
				27.5–29.9	0.79 (0.71-0.87)	<.0001	
				≥ 30.0	0.93 (0.78-1.12)	.45	
			Adjusted HR (women)	16.0–17.4	2.94 (2.57-3.37)	<.0001	Yes
				17.5–19.9	1.94 (1.79-2.09)	<.0001	
				20.0–22.4	1.29 (1.21-1.38)	<.0001	
				22.5–24.9	Reference		
				25.0–27.4	0.84 (0.78-0.91)	<.0001	
				27.5–29.9	0.89 (0.81-0.97)	.01	
				≥ 30.0	0.92 (0.80-1.05)	.21	
Lee, 2018 [56]	60-month mortality	None specific	Adjusted HR	<18.5	2.58 (2.24-2.97)	<.05	No
				18.5–22.9	1.41 (1.29-1.55)	<.05	
				23.0–24.9	Reference		
				25.0–27.4	1.02 (0.91-1.14)	NS	
				27.5–29.9	0.92 (0.77-1.09)	NS	
				≥ 30.0	1.12 (0.85-1.47)	NS	

Lv, 2018 [23]	36-month mortality	None specific	Adjusted HR	<18.5	Reference		Yes
				18.5–22.0	0.71 (0.60-0.83)	<0.01	
				≥ 24.0	0.60 (0.47-0.76)	<0.01	
de Souto Barreto, 2017 [24]	18-month mortality	Dementia	Adjusted HR (With dementia)	<18.5	1.61 (1.25–2.09)	<.0001	Yes
				18.5–24.9	Reference		
				25.0–29.9	0.60 (0.49–0.75)	<.0001	
				≥ 30.0	0.54 (0.38–0.76)	<.0001	
			Adjusted HR (Without dementia)	<18.5	1.19 (0.87–1.64)	.28	No
				18.5–24.9	Reference		
				25.0–29.9	0.81 (0.65–1.00)	.05	
				≥ 30.0	0.77 (0.60–1.00)	.05	
Wu, 2017 [25]	12-month mortality	Atrial fibrillation	Adjusted HR (65-74 years)	<18.5	1.56 (0.69–3.51)	NS	No
				18.5–23.9	Reference		
				24.0–27.9	0.29 (0.13–0.62)	<.05	
				≥ 28.0	0.61 (0.23–1.60)	NS	
			Adjusted HR (≥ 75 years)	<18.5	0.95 (0.59–1.52)	NS	Yes
				18.5–23.9	Reference		
				24.0–27.9	0.69 (0.47–0.99)	<.05	
				≥ 28.0	0.46 (0.22–0.95)	<.05	
Cheng, 2016 [57]	132-month mortality	None specific	Adjusted HR	<18.5	3.35 (1.88-5.96)	<.05	Yes
				18.5–24.9	Reference		
				25.0–29.9	0.80 (0.70-0.90)	<.05	
				30.0–34.9	.078 (0.69-0.89)	<.05	

				35.0–39.9	0.96 (0.81-1.12)	NS	
				≥ 40.0	1.17 (0.93-1.47)	NS	
		Diabetes		<18.5	-		Yes
				18.5–24.9	Reference		
				25.0–29.9	0.75 (0.61–0.93)	<.05	
				30.0–34.9	0.78 (0.63–0.96)	<.05	
				35.0–39.9.9	0.90 (0.70–1.15)	NS	
				≥ 40.0	1.09 (0.79–1.49)	NS	
		Hypertension		<18.5	2.75 (1.36–5.56)	<.05	Yes
				18.5–24.9	Reference		
				25.0–29.9	0.82 (0.72–0.93)	<.05	
				30.0–34.9	0.80 (0.70–0.92)	<.05	
				35.0–39.9.9	0.98 (0.83–1.17)	NS	
				≥ 40.0	1.13 (0.89–1.44	NS	
		Dyslipidaemia		<18.5	2.74 (1.36–5.53	<.05	
				18.5–24.9	Reference		
				25.0–29.9	0.81 (0.72–0.91)	<.05	
				30.0–34.9	0.81 (0.71–0.92)	<.05	
				35.0–39.9.9	1.02 (0.86–1.20)	NS	
				≥ 40.0	1.21 (0.96–1.53)	NS	
Flodin, 2016 [26]	12-month survival	Hip fracture	Adjusted OR	<22.0	Reference		Yes
				22.0–26.0	0. 90 (0.60–1.40)	.60	
				> 26.0	2.60 (1.20–5.50)	.01	

Calabia, 2015 [58]	120-month mortality	Haemodialysis	Adjusted HR	<18.0	2.23 (1.87–2.66)	<.001	Yes
				18.0–19.9	1.76 (1.51–2.05)	<.001	
				20.0–21.4	1.47 (1.26–1.71)	<.001	
				21.5–22.9	1.32 (1.14–1.52)	<.001	
				23.0–24.9	Reference		
				25.0–27.4	1.03 (0.89–1.18)	NS	
				27.5–29.9	0.93 (0.79–1.10)	NS	
				30.0–34.9	0.76 (0.62–0.93)	<.001	
				≥ 35.0	0.77 (0.54–1.10)	NS	
Kim, 2015 [59]	108-month mortality	Chronic diseases	Adjusted HR	<18.5	2.07 (1.56–2.75)	<.05	No
				18.5–19.9	1.56 (1.20–2.02)	<.05	
				20.0–21.4	1.22 (0.96–1.55)	NS	
				21.5–22.9	1.21 (0.97–1.52)	NS	
				23.0–24.9	Reference		
				25.0–26.4	0.90 (0.69–1.18)	NS	
				26.5–27.9	1.06 (0.76–1.47)	NS	
				28.0–29.9	0.84 (0.56–1.27)	NS	
				30.0–32.4	0.81 (0.42–1.56)	NS	
				≥ 32.5	1.58 (0.63–3.99)	NS	
Kubota, 2015 [60]	132-month ID mortality	Diabetes mellitus	Adjusted HR	<20.0	1.25 (0.71–2.20)	NS	Yes
				20.0–22.9	Reference		
				23.0–24.9	0.55 (0.30–1.03)	NS	
				≥ 25.0	0.54 (0.30–0.98)	<.05	

Kuo, 2015 [27]	66-month mortality	Diabetes mellitus	Unadjusted HR*	17.5–19.9	1.80	<.001	No
				20.0–22.4	1.20	NS	
				22.5–24.9	Reference		
				25.0–27.4	0.95	NS	
				27.5–29.9	0.79	NS	
				30.0–32.5	1.62	<.001	
				32.5–35.0	2.58	<.001	
Shil Hong, 2015 [61]	72-month mortality	None specific	Adjusted HR	15.3–21.7	Reference	.004	Yes
				21.8–23.7	0.76 (0.53–1.10)		
				23.8–25.4	0.63 (0.41–0.96)		
				25.5–33.7	0.54 (0.34–0.86)		
Buys, 2014 [28]	102-month mortality	None specific	Adjusted HR	<18.5	1.57 (0.88-2.82)	NS	No
				18.5–24.9	Reference		
				25.0–29.9	0.94 (0.73-1.21)	NS	
				30.0–34.9	0.80 (0.58-1.09)	NS	
				≥ 35.0	0.73 (0.49-1.09)	NS	
Clark, 2014 [62]	120-month mortality	None specific	Adjusted HR (Africans)	<18.5	1.34 (1.11–1.63)	<.05	No
				18.5–24.9	Reference		
				25.0–29.9	0.81 (0.61–1.07)	NS	
				≥ 30.0	0.82 (0.54–1.24)	NS	
			Adjusted HR (African	<18.5	2.59 (1.44–4.65)	<.05	No
				18.5–24.9	Reference		
				25.0–29.9	0.91 (0.72–1.15)	NS	

			Americans)	≥ 30.0	1.04 (0.83–1.31)	NS	
Ford, 2014 [29]	40-month mortality	None specific	Adjusted HR	<18.5	1.47 (0.84-547)	.18	No
				18.5–24.9	Reference		
				25.0–29.9	0.78 (0.60-1.00)	.05	
				30.0–34.9	0.80 (0.59-1.10)	.17	
				≥ 35.0	0.75 (0.48-1.17)	.20	
Lang, 2014 [30]	6-week mortality	None specific	Adjusted HR	<18.0	1.80 (1.00–3.00)	.04	Yes
				18.0–24.9	Reference		
				25.0–29.9	0.90 (0.70–1.40)	.77	
				≥ 30.0	0.50 (0.30–0.90)	.02	
	12-month mortality		Adjusted HR	<18.0	2.00 (1.50–2.70)	<.001	Yes
				18.0–24.9	Reference		
				25.0–29.9	0.80 (0.70–1.00)	.04	
				≥ 30.0	0.70 (0.50–0.90)	.002	
	24-month mortality		Adjusted HR	<18.0	1.80 (1.30–2.30)	<.001	Yes
				18.0–24.9	Reference		
				25.0–29.9	0.80 (0.70–1.00)	.04	
				≥ 30.0	0.70 (0.60–0.90)	.002	
Lee, 2014 [31]	36-month mortality	None specific	Adjusted HR	<18.5	2.04 (1.55-2.70)	<.0001	Yes
				18.5–24.9	Reference		
				25.0–29.9	0.74 (0.57-0.96)	<.05	
				≥ 30.0	1.01 (0.57-1.79)	NS	
Murphy, 2014 [63]	84-month	Diabetes	Adjusted	18.5–24.9	1.72 (1.12–2.64)	<.05	No

	mortality	mellitus	HR	25.0–29.9	Reference		
				≥ 30.0	0.89 (0.58–1.38)	NS	
Wu, 2014 [32]	60-month mortality	None specific	Adjusted HR	15.0–18.4	1.92 (1.71-2.15)	<.05	Yes
				18.5–24.9	Reference		
				25–29.9	0.82 (0.76-0.88)	<.05	
				30–34.9	0.82 (0.69-0.98)	<.05	
				≥ 35	1.59 (1.06-2.38)	<.05	
	60-month CVD mortality		Adjusted HR	15.0–18.4	1.74 (1.36-2.23)	<.05	Yes
				18.5–24.9	Reference		
				25–29.9	0.85 (0.73-0.99)	<.05	
				30–34.9	0.75 (0.51-1.09)	NS	
				≥ 35	2.36 (1.17-4.76)	<.05	
Yamauchi, 2014 [64]	In-hospital mortality	COPD	Adjusted OR	<18.5	1.55 (1.48–1.38)	<.0001	Yes
				18.5–22.9	Reference		
				23.0–24.9	0.76 (0.70–0.82)	<.0001	
				25.0–29.9	0.73 (0.66–0.80)	<.0001	
				≥ 30.0	0.67 (0.52–0.86)	.002	
Chen, 2013 [33]	18-month mortality	None specific	Adjusted HR	<18.5	Reference		Yes
				18.5–22.9	1.93 (1.16-3.20)	<.05	
				23.0–24.9	0.56 (0.36-0.87)	<.05	
Dahl, 2013 [34]	216-month mortality	None specific	Adjusted HR	< 25.0	Reference		Yes
				25.0–29.9	0.80 (0.67-0.95)	<.05	
				≥ 30.0	0.93 (0.71-1.22)	NS	

Nakazawa, 2013 [35]	12-month mortality	None specific	Adjusted HR	<17.3	2.40 (1.90-3.10)	<.05	Yes
				17.3–19.2	1.70 (1.30-2.30)	<.05	
				19.3–21.1	1.50 (1.20-2.00)	<.05	
				21.2–23.5	1.20 (0.90-1.60)	NS	
				≥ 23.6	Reference		
Takata, 2013 [36]	144-month mortality	None specific	Adjusted HR	< 19.5	1.34 (0.93-1.94)	NS	Yes
				19.5–21.1	1.05 (0.72-1.52)	NS	
				21.1–22.5	Reference		
				22.5–23.8	0.63 (0.42-0.94)	<.05	
				23.8–26.0	0.73 (0.50-1.06)	NS	
				≥ 26.0	0.82 (0.56-1.21)	NS	
	144-month CVD mortality		Adjusted HR	< 19.5	1.40 (0.67-2.90)	NS	No
				19.5–21.1	1.01 (0.51-2.22)	NS	
				21.1–22.5	Reference		
				22.5–23.8	0.64 (0.29-1.41)	NS	
				23.8–26.0	0.85 (0.42-1.75)	NS	
				≥ 26.0	1.27 (0.64-2.53)	NS	
	144-month cancer mortality		Adjusted HR	< 19.5	1.46 (0.54-3.93)	NS	No
				19.5–21.1	1.90 (0.76-4.75)	NS	
				21.1–22.5	Reference		
				22.5–23.8	0.91 (0.32-2.63)	NS	
				23.8–26.0	1.59 (0.64-3.94)	NS	
				≥ 26.0	0.87 (0.30-2.51)	NS	

Tseng, 2013 [37]	144-month mortality	Diabetes mellitus	Unadjusted rate ratio#	<18.5	1.48 (1.39–1.58)	<.0001	Yes
				18.5–22.9	Reference		
				23.0–24.9	0.71 (0.69–0.74)	<.0001	
				25.0–29.9	0.66 (0.63–0.68)	<.0001	
				≥ 30.0	0.68 (0.63–0.73)	<.0001	
Veronese, 2013 [38]	60-month mortality	None specific	Adjusted HR	< 20	1.44 (0.95-3.60)	.25	Yes
				20–24.9	Reference		
				25.0–29.9	0.90 (0.61-1.71)	.70	
				≥ 30.0	0.43 (0.20-0.70)	.01	
Woo, 2013 [39]	84-month mortality	None specific	Adjusted HR	<21.6	1.37 (1.00–1.88)	.04	No
				21.6–23.5	Reference		
				23.5–25.7	1.29 (0.95–1.73)	.10	
				≥25.7	1.53 (1.02–2.29)	.04	
Yamamoto, 2013 [40]	30-day mortality	TAVI patients	Adjusted HR	<18.5	1.54 (0.87–2.74)	.14	No
				18.5–24.9	Reference		
				25.0–29.9	0.77 (0.58–1.03)	.08	
				≥ 30.0	0.71 (0.49–1.03)	.07	
	12-month mortality		Adjusted HR	<18.5	1.25 (0.78–2.00)	.35	Yes
				18.5–24.9	Reference		
				25.0–29.9	0.81 (0.66–1.00)	.05	
				≥ 30.0	0.74 (0.57–0.97)	.03	
Zekry, 2013 [41]	48-month mortality	None specific	Adjusted HR	< 20	1.07 (0.73-1.53)	.75	Yes
				20.0–24.9	Reference		

				25.0–29.9	0.78 (0.55-1.10)	.16	
				≥ 30.0	0.52 (0.31-0.88)	.02	
de Hollander, 2012 [42]	120-month mortality	None specific	Adjusted HR	< 20	1.06 (0.73-1.55)	NS	No
				20.0–24.9	Reference		
				25.0–29.9	0.92 (0.78-1.09)	NS	
				≥ 30.0	1.05 (0.89-1.29)	NS	
Kvamme, 2012 [43]	12-month mortality	None specific	Adjusted HR (men)	< 18.5	2.32 (1.75-3.07)	<.05	No
				18.5–19.9	1.28 (1.03-1.60)	<.05	
				20.0–22.4	1.23 (1.09-1.38)	<.05	
				22.5–24.9	1.12 (1.02-1.22)	<.05	
				25.0–27.4	Reference		
				27.5–29.9	1.05 (0.95-1.15)	NS	
				30.0–32.4	1.19 (1.05-1.34)	<.05	
				32.5–34.9	1.31 (1.09-1.56)	<.05	
				≥ 35	1.53 (1.21-1.95)	<.05	
			Adjusted HR (women)	< 18.5	1.49 (1.06-2.10)	<.05	No
				18.5–19.9	1.03 (0.78-1.36)	NS	
				20.0–22.4	1.11 (0.97-0.88)	NS	
				22.5–24.9	0.97 (0.88-1.06)	NS	
				25.0–27.4	Reference		
				27.5–29.9	1.04 (0.94-1.14)	NS	
				30.0–32.4	1.16 (1.03-1.31)	<.05	
				32.5–34.9	1.43 (1.20-1.72)	<.05	

		≥ 35	1.53 (1.24-1.90)	<.05	
12-month RD mortality	Adjusted HR (men)	< 20.0	4.35 (2.87-6.60)	<.05	No
		20.0–22.4	2.45 (1.76-3.40)	<.05	
		22.5–24.9	1.58 (1.19-2.10)	<.05	
		25.0–27.4	Reference		
		27.5–29.9	0.81 (0.57-1.16)	NS	
		≥ 30	1.18 (0.81-1.71)	NS	
	Adjusted HR (women)	< 20.0	3.30 (2.07-5.27)	<.05	No
		20.0–22.4	1.83 (1.21-2.75)	<.05	
		22.5–24.9	1.32 (0.91-1.91)	NS	
		25.0–27.4	Reference		
		27.5–29.9	0.84 (0.54-1.28)	NS	
		≥ 30	1.06 (0.73-1.55)	NS	
12-month CVD mortality	Adjusted HR (men)	< 20.0	1.14 (0.84-1.54)	NS	No
		20.0–22.4	0.98 (0.82-1.19)	NS	
		22.5–24.9	1.07 (0.94-1.22)	NS	
		25.0–27.4	Reference		
		27.5–29.9	1.12 (0.98-1.28)	NS	
		≥ 30	1.33 (1.14-1.54)	<.05	
	Adjusted HR (women)	< 20.0	1.28 (0.98-1.67)	NS	No
		20.0–22.4	1.21 (1.01-1.44)	<.05	
		22.5–24.9	1.01 (0.98-1.32)	NS	
		25.0–27.4	Reference		

				27.5–29.9	1.14 (0.98-1.32)	NS	
				≥ 30	1.09 (0.95-1.26)	NS	
	12-month cancer mortality		Adjusted HR (men)	< 20.0	1.43 (0.99-2.07)	NS	No
				20.0–22.4	1.22 (0.98-1.54)	NS	
				22.5–24.9	1.17 (0.99-1.54)	NS	
				25.0–27.4	Reference		
				27.5–29.9	1.12 (0.94-1.42)	NS	
				≥ 30	1.16 (0.95-1.42)	NS	
			Adjusted HR (women)	< 20.0	1.49 (1.03-2.15)	<.05	No
				20.0–22.4	1.16 (0.89-1.53)	NS	
				22.5–24.9	1.12 (0.89-1.40)	NS	
				25.0–27.4	Reference		
				27.5–29.9	0.99 (0.78-1.25)	NS	
				≥ 30	1.28 (1.04-1.57)	<.05	
Mihel, 2012 [44]	60-month mortality	Hypertension	Unadjusted rate (%) (Men)	<25.0	31.2 (25.5–36.9)	Ref.	Yes
				25.0–29.9	23.6 (19.3–27.8)	NS	
				≥ 30.0	15.4 (9.9–21.0)	<.05	
			Unadjusted rate (%) (Women)	<25.0	24.6 (20.9–28.4)	Ref.	No
				25.0–29.9	18.5 (15.6–21.4)	NS	
				≥ 30.0	18.4 (15.0–21.8)	NS	
Tsai, 2012 [65]	48-month mortality (65-74 years)	None specific	Adjusted HR (Men)	<21.0	2.06 (1.39-3.04)	<.001	No
				21.0–27.0	Reference		
				> 27.0	0.60 (0.27-1.31)	NS	

			Adjusted HR (Women)	<21.0<br>21.0–27.0<br>> 27.0	1.76 (1.00-3.13)<br>Reference<br>0.62 (0.26-1.49)	<.05<br><br>NS	No
	48-month mortality (≥ 75 years)		Adjusted HR (Men)	<21.0<br>21.0–27.0<br>> 27.0	1.54 (1.15–2.06)<br>Reference<br>0.57 (0.29–1.12)	<.01<br><br>NS	No
			Adjusted HR (Women)	<21.0<br>21.0–27.0<br>> 27.0	2.05 (1.44–2.91)<br>Reference<br>0.83 (0.46–1.52)	<.001<br><br>NS	No
Cereda, 2011 [45]	73-month mortality	None specific	Adjusted HR	< 21.0<br>21.0–24.9<br>≥ 25.0	1.53 (1.13-2.06)<br>1.04 (0.77-1.40)<br>Reference	.006<br>.79	Yes
Berraho, 2010 [46]	156-month mortality	None specific	Adjusted HR	< 18.5<br>18.5–21.9<br>22.0–24.9<br>25.0-29.9<br>≥ 30	1.45 (1.17-1.78)<br>1.27 (1.12-1.43)<br>Reference<br>0.98 (0.88-1.10)<br>1.06 (0.89-1.27)	<.05<br><.05<br><br>NS<br>NS	No
Han, 2010 [47]	42-month mortality	None specific	Adjusted HR	< 22.0<br>22.0–26.1<br>≥ 26.1	Reference<br>0.93 (0.50-1.72)<br>0.54 (0.22-1.36)	 .81<br>.19	No
Kitamura, 2010 [48]	24-month mortality	None specific	Adjusted OR	<17.1<br>≥ 17.1	3.96 (1.79-8.79)<br>Reference	.0007	Yes
Lea, 2009 [66]	125-month	AMI	Adjusted	<18.5	1.45 (1.40–1.51)	<.0001	Yes

	mortality		HR	18.5–24.9	Reference		
				25.0–29.9	0.85 (0.83–0.87)	<.0001	
				30.0–34.9	0.85 (0.83–0.87)	<.0001	
				35.0–39.9	0.89 (0.84–0.93)	<.0001	
				≥ 40.0	0.95 (0.88–1.01)	.11	
Luchsinger, 2008 [49]	144-month mortality	None specific	Adjusted HR	<23.0	1.60 (1.20-2.00)	<.05	No
				23.0–26.1	Reference		
				26.2–29.4	1.10 (0.80-1.40)	NS	
				≥ 29.4	1.10 (0.80-1.40)	NS	
Locher, 2007 [50]	36-month mortality	None specific	Adjusted HR	< 18.5	2.18 (1.02-4.66)	.45	No
				18.5–24.9	Reference		
				25.0–29.9	1.25 (0.83-1.87)	.28	
				30.0-34.9	0.83 (0.49-1.42)	.50	
				≥ 35	0.92 (0.47-1.80)	.81	
Takata, 2007 [51]	48-month mortality	None specific	Adjusted HR	< 18.5	1.94 (1.00-3.76)	<.05	Yes
				18.5–24.9	Reference		
				≥ 25.0	0.48 (0.24-0.96)		
	48-month CVD mortality			< 18.5	4.64 (1.68-12.80	<.05	Yes
				18.5–24.9	Reference		
				≥ 25.0	1.02 (0.35-2.95)		
	48-month cancer mortality			< 18.5	2.17 (0.60-7.85)	NS	No
				18.5–24.9	Reference		
				≥ 25.0	0.26 (0.06-1.20)		

Grabowski, 2001 [67]	96-month mortality	None specific	Adjusted HR	< 19.4	1.58 (1.41-1.78)	<.0001	Yes
				19.5–28.4	Reference		
				≥ 28.5	0.83 (0.74-0.94)	.002	

HR/OR: Hazard ratio / Odds ratio; ID: Infectious diseases

* 95% CI were not computed

# The estimates and their respective confidence intervals were not presented in the original articles, but were calculated with the information available in these articles.

TAVI: Transcatheter Aortic Valve Implementation; AHF: Acute heart failure; COPD: Chronic Obstructive Pulmonary Disease; AMI: Acute Myocardial Infarction; RD: Respiratory disease; CVD: cardiovascular disease.
